# Supplementary material for: Reconstitution of the complete pathway of ITS2 processing at the pre-ribosome
Source: Nat Commun. 2017 Nov 27;8:1787. doi: 10.1038/s41467-017-01786-9 (PMC5702609; doi:10.1038/s41467-017-01786-9)

SUPPLEMENTARY INFORMATION

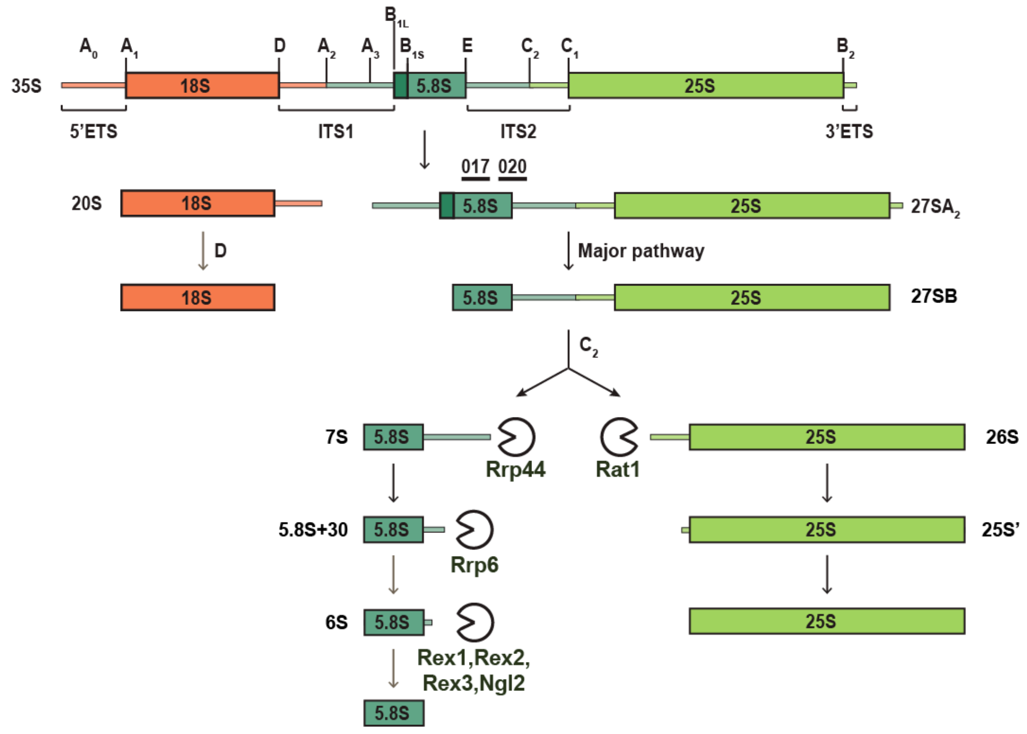

Supplementary Figure 1 Scheme depicting processing of yeast 35S pre-rRNA to yield mature 5.8S and 25S rRNA. The 35S precursor consists of a 5' external transcribed spacer (5'ETS), 18S rRNA, internal transcribed spacer 1 (ITS1), 5.8S rRNA, internal transcribed spacer 2 (ITS2), 25S rRNA and 3' external transcribed spacer (3'ETS). The cleavage sites within the 35S pre-rRNA are indicated. For 5.8S rRNA maturation, only the major pathway is depicted ($5.8S_S$ as opposed to $5.8S_L$), but the removal of ITS2 is the same in both cases. 27SB pre-rRNA is cleaved at site C_2 by the Las1 endonuclease. The resulting cleaved products, 7S and 26S pre-rRNA, are further processed through different intermediates (5.8S+30, and 6S or 25S' pre-rRNA) to yield mature 5.8S and 25S rRNA (7S→5.8S versus 26S→25S pre-rRNA processing). The different ribonucleases involved are indicated at their respective processing steps. 7S pre-rRNA is processed by the Rrp44 exosome subunit until the 5.8S+30 intermediate, which is processed by the other nucleolytic exosome subunit, Rrp6, to yield 6S pre-rRNA that is only extended by eight nucleotides relative to the mature 5.8S rRNA. These eight nucleotides are removed after nuclear export by cytoplasmic exonucleases.

The 26S pre-rRNA is processed by Rat1 5'→3' exonuclease to 25S rRNA via a 25S' intermediate. Above the $27SA_2$ pre-rRNA the location of the probes used for detection in the northern blots is indicated.

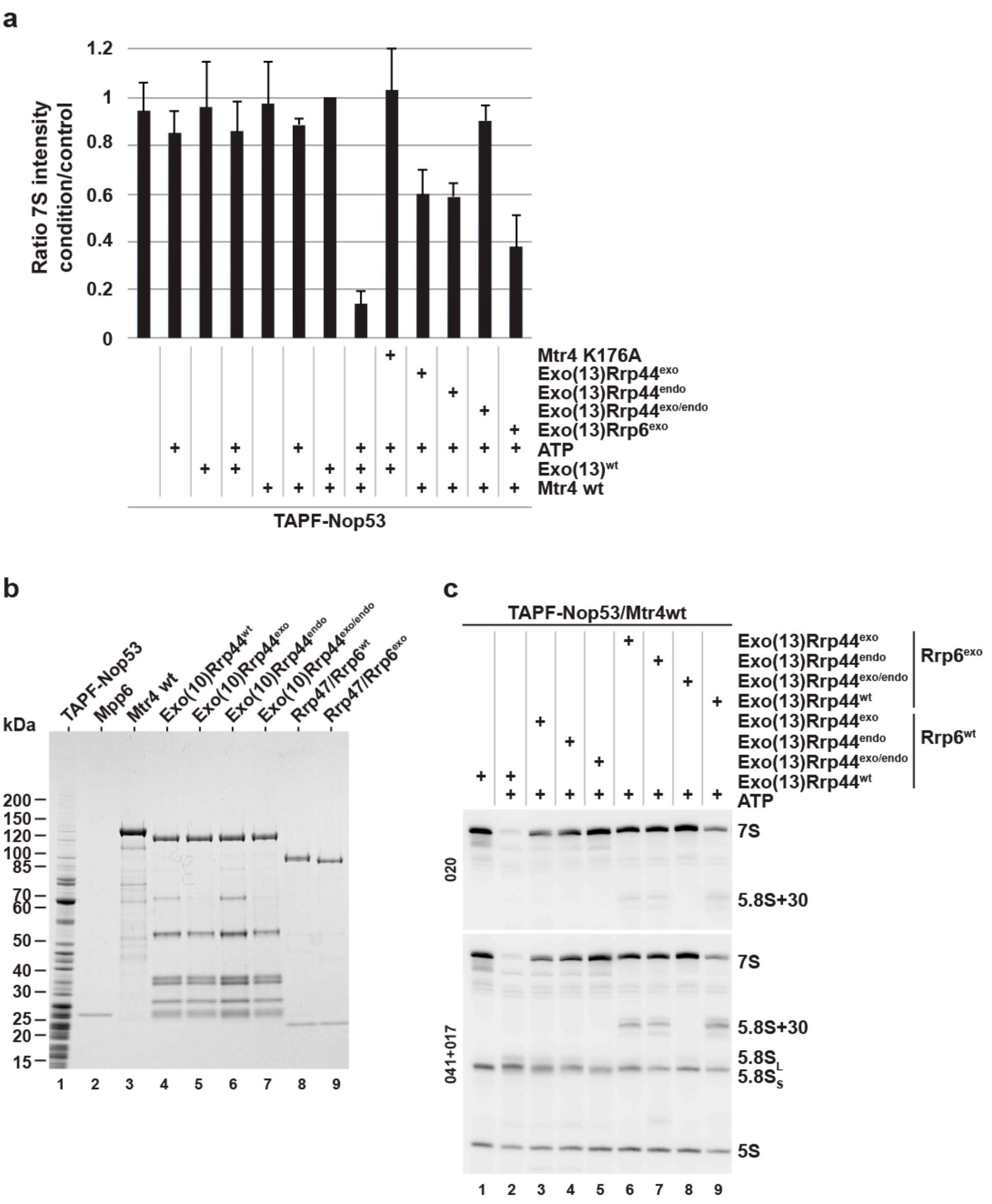

Supplementary Figure 2 Pre-rRNA maturation in the context of the *in vitro* 7S→5.8S pre-rRNA processing assay using wt or mutant exosome. (**a**) Quantification of 7S pre-rRNA bands in **Figure 2b** and two more experiments (n=3). Background was subtracted and bands were normalized to 5S rRNA levels. 7S levels of the condition with all necessary factors but without ATP (control) were set to one. Error bars correspond to one standard deviation. (**b**) SDS-PAGE and Coomassie staining of the protein samples used for *in vitro* 7S→5.8S pre-rRNA processing shown in (**c**). Pre-60S particles containing 7S substrate pre-rRNA were affinity-purified via TAPF-Nop53. All the yeast exosome subunits and Mtr4 were expressed recombinantly in *E. coli* and

subsequently purified from cell lysates. (**b**) Northern blot for the detection of 7S pre-rRNA, 5.8S rRNA and 5S rRNA species after the *in vitro* processing reaction. Processing of 7S pre-rRNA from pre-60S particles (TAPF-Nop53) using the recombinantly purified exosome (Exo(13)) and its cofactor Mtr4, either the wt (wt) or K176A mutant, in the presence or absence of ATP. Exo(13) was used combining mutations in Rrp44 with the Rrp6 wt or the Rrp6 exonucleolytic mutant. 7S pre-rRNA was detected with two different probes. 5S rRNA served as a loading control.

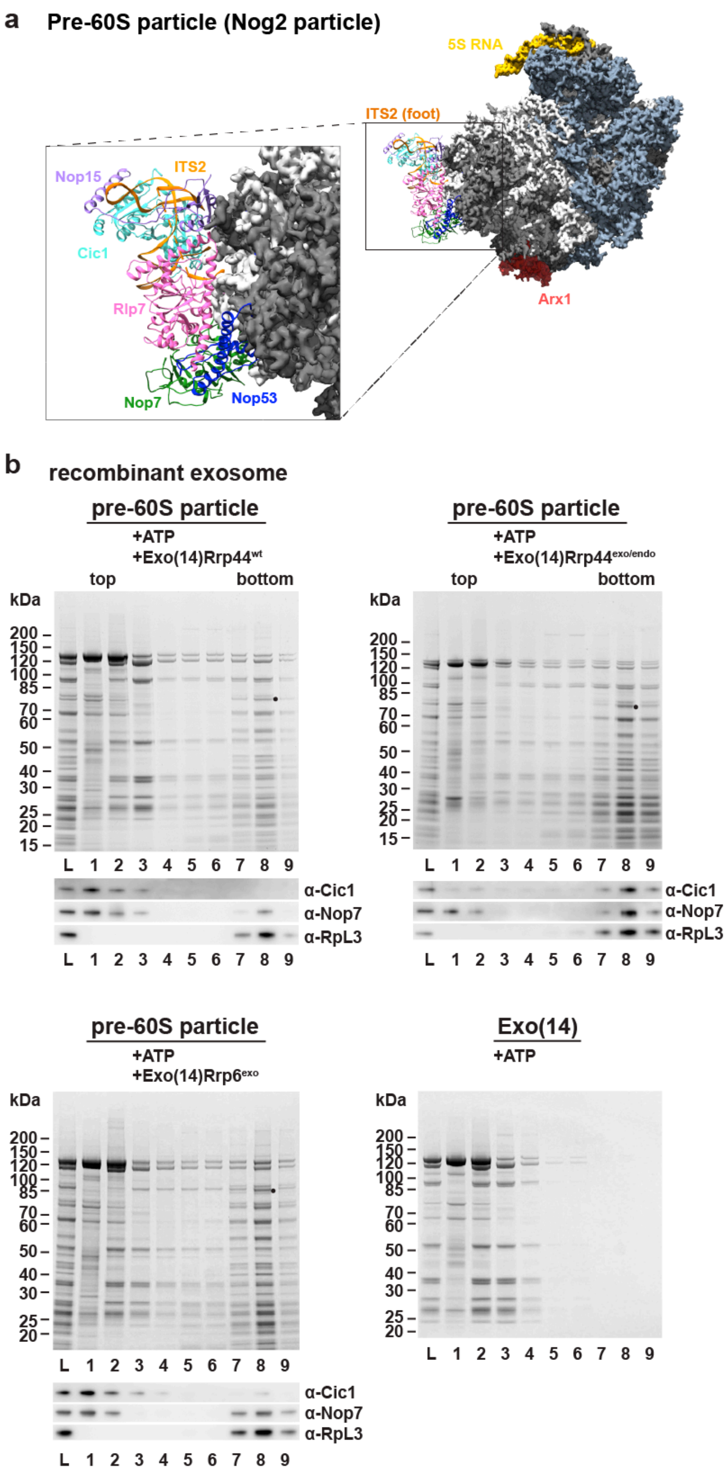

Supplementary Figure 3 Analysis of pre-60S particles incubated with recombinant exosome reveals release of ITS2 associated factors after *in vitro* 7S→5.8S pre-rRNA processing. (**a**) Published cryo-EM structure of the Nog2-derived pre-60S ribosomal particle (Wu et al., 2016) indicating details of the prominent "foot" structure. Shown are: r-proteins (dark grey), 25S and 5.8S rRNA (light grey), Arx1 (red), 5S rRNA (yellow), part of ITS2 (orange), Nop15

(purple), Cic1 (cyan), Rlp7 (pink), Nop7 (green), Nop53 (blue), residual ribosome biogenesis factors (blue-grey). (**b**) Glycerol gradient centrifugation of recombinantly expressed (in *E. coli)* exosome (Exo14) and Nop53-derived pre-60S particles incubated with the indicated reagents (ATP, Exo14). Gradient fractions were collected, precipitated by TCA and analysed by SDS-PAGE and Coomassie staining (upper panels) or by western blotting using the indicated antibodies (lower panels). Results are shown for a pre-60S particle incubated with recombinant exosome containing all wt components (upper left) or mutant components in the presence of ATP (upper right: Rrp44 double mutant; lower left: Rrp6 exonuclease mutant). Exo(14) includes all components of the nuclear exosome: the core, the exonucleases and cofactors, as well as Mtr4. Fractions (7–9) contained the pre-60S particles, whereas the top fractions (1 and 2) contained the released factors such as Cic1 and Nop7. Fractions 2–4 contained exosome. Ribosome assembly factor Nog1 not released during the *in vitro* reaction is indicated by a dot (fraction 8).

a **pre-60S particle (TAPF-Nop53)**

+ATP

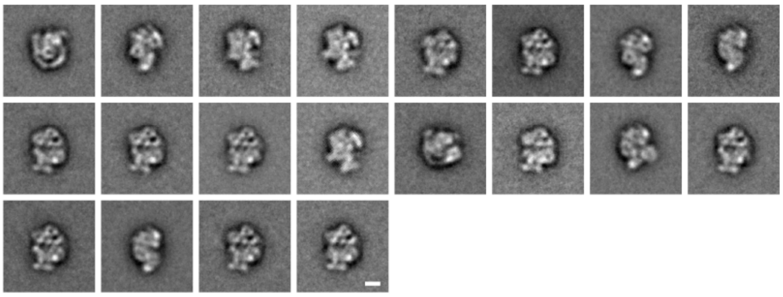

b **pre-60S particle (TAPF-Nop53)**

+ATP
+exosome (Rrp6-TAP)
+Mtr4

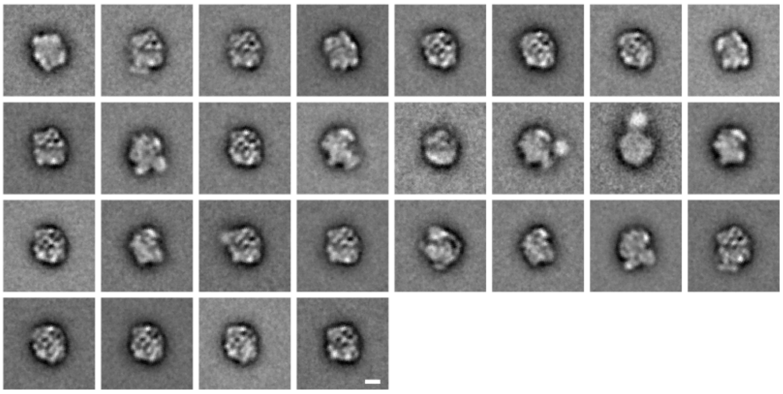

c **pre-60S particle (TAPF-Nop53)**

+ATP
+exosome (Rrp6-TAP)
+Mtr4 K176A

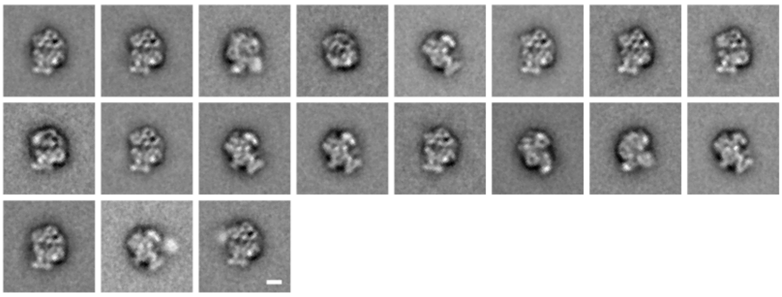

Supplementary Figure 4 Detection of “foot” structure removal from pre-60S particles (related to to **Figure 4**). Negative-stain EM 2D averages of pre-60S particles (affinity-purified via TAPF-Nop53) after the *in vitro* processing reaction. (**a**) pre-60S particles treated with only ATP as a control (mock). Averages of 3908 particles are shown. (**b**) pre-60S particles treated with endogenous yeast exosome (affinity-purified via Rrp6-FTpA) and recombinant wt Mtr4 in the presence of ATP. Averages of 4414 particles are shown. (**c**) pre-60S particles treated with endogenous yeast exosome (affinity-purified via

Rrp6-FTpA) and recombinant mutant Mtr4 (K176A) in the presence of ATP. Averages of 3870 particles are shown. The pre-60S particles shown were derived from fraction 8 of the glycerol gradient (**Fig. 3a**). The scale bars represent 10 nm.

Supplementary Table 1 Yeast strains used in this study

Name	Genotype	Source
W303	*Mat*α*, ade2-1, ura3-1, leu2-3,112, his3-11,15, trp1-1, can1-100*	Thomas and Rothstein, 1989
TAP-Flag-Nop53	W303, *Mat*α*,* P_{NOP53}*-TAP-Flag-NOP53::natNT2*	Thoms et al., 2015
Rrp6-TAP	W303, *Mat*α, *RRP6-FTpA::natNT2*	Gasse et al., 2015
Rsa4-TAP/ Las1-AID	W303, *Mat*α*,* P_{ADH1}*-OsTIR1-9xmyc::Trp1, TAP-Flag-RSA4::natNT2, LAS1-HA-AID::His3MX6*	Gasse et al., 2015

Supplementary Table 2 Plasmids used in this study

Name	Features	Source
pET21d-His-TEV-Mtr4	*AmpR, 6xHis-TEV-Mtr4*	This study
pET21d-His-TEV-Mtr4 K176A	*AmpR, 6xHis-TEV-Mtr4 K176A*	This study
YEplac112-P2-Las1-TpA-p.Gal1-10-P1-Flag-Grc3	*2*μ*, TRP1, P2-LAS1-TpA-*$P_{GAL1-10}$ *-P1-Flag-Grc3*	Gasse et al., 2015
YEplac112-P2-Las1-TpA-p.Gal1-10-P1-Flag-Grc3(K252A)	*2*μ*, TRP1, P2-LAS1-TpA-*$P_{GAL1-10}$*-P1-Flag-Grc3(K252A)*	Gasse et al., 2015
YEplac181-P2-Rat1-p.Gal 1-10-P1-Rai1	*2*μ*, LEU2, P2-Rat1-*$P_{GAL1-10}$*-P1-Rai1*	Gasse et al., 2015

Supplementary Table 3 LFQ intensity values of fractions 8 of two experiments normalized to Nog1 (filtered for ribosomal proteins, contaminations and exosome components and cofactors)

	**Experiment 1**			**Experiment 2**		
**Protein IDs**	**LFQ intensity wt**	**LFQ intensity Rrp44**	**LFQ intensity Rrp6**	**LFQ intensity wt**	**LFQ intensity Rrp44**	**LFQ intensity Rrp6**
**Cic1**	155540000	1271726242	296606391.8	617450000	5906540191	1543663069
**Nop2**	26340000	162218082.9	36305954.21	195130000	775687561.3	205926273.6
**Rlp7**	229290000	1151957424	311031247.3	1261100000	6436868387	2460270181
**Nop15**	45031000	209770601.4	67193189.44	140250000	1007385757	384648327.5
**Spb1**	41179000	150540156	35384396.06	147620000	383754678.1	82485493.64
**Nop53**	835430000	1653711232	1281373983	3281600000	8047010322	7217219366
**Nop7**	911070000	1668308641	1473311561	5507000000	9083885180	9121253413
**Bud20**	169360000	235789022.6	217113945.5	2920600000	2523484938	2942419895
**Nsa2**	739210000	758025914.7	824987881.5	4486400000	3740703092	4190651933
**Nog2**	2144300000	2149439230	2135480095	14666000000	13714017623	17920551028
**Nog1**	2627300000	2627300000	2627300000	12250000000	12250000000	12250000000
**Nug1**	2089100000	2010004783	2155565337	10265000000	9634559799	12467032703
**Mrt4**	973590000	930497217.5	967904580.8	6435600000	6034564113	6683588083
**Arx1**	3707400000	3534441364	4242282331	21152000000	20244690948	23715494069
**Ipi3**	715710000	678814536.4	824998622.3	4053000000	3189103636	3987861592
**Tif6**	396410000	374219168.4	457771661	3704700000	3336813382	4539132499
**Alb1**	250040000	227637829.6	212409487.8	2143500000	1828535683	2823777093
**Rsa4**	1586600000	1423422513	1689523282	9062500000	8177544516	9514682353
**Ipi1**	154340000	135522342	135913716.5	979800000	935552314	1090690599
**Rix1**	935770000	730150703.2	964456793.3	4244900000	3443169685	4231288399
**Rrs1**	240590000	183273385.2	249637000.1	1744000000	1564824901	1666225787
**Sda1**	750480000	559010684.1	791261860.5	4046100000	3390850311	3614162898
**Rpf2**	412340000	231433155.8	407375963.4	1649900000	1502728675	1907169980

Supplementary Figure 5 Uncropped scans of all gels and blots of the main text

related to Fig. 1 a

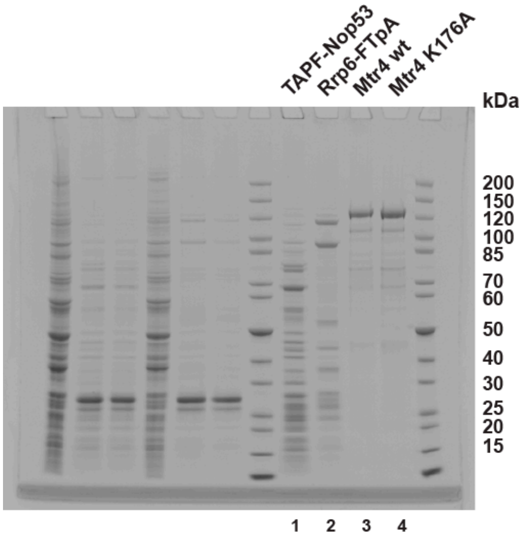

related to Fig. 1 b

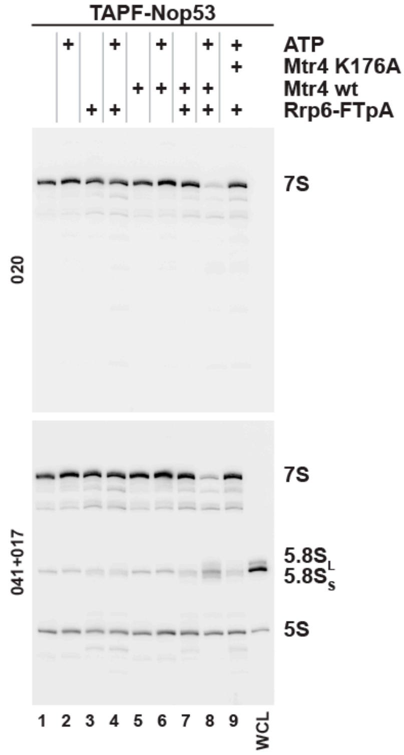

related to Fig. 2 b

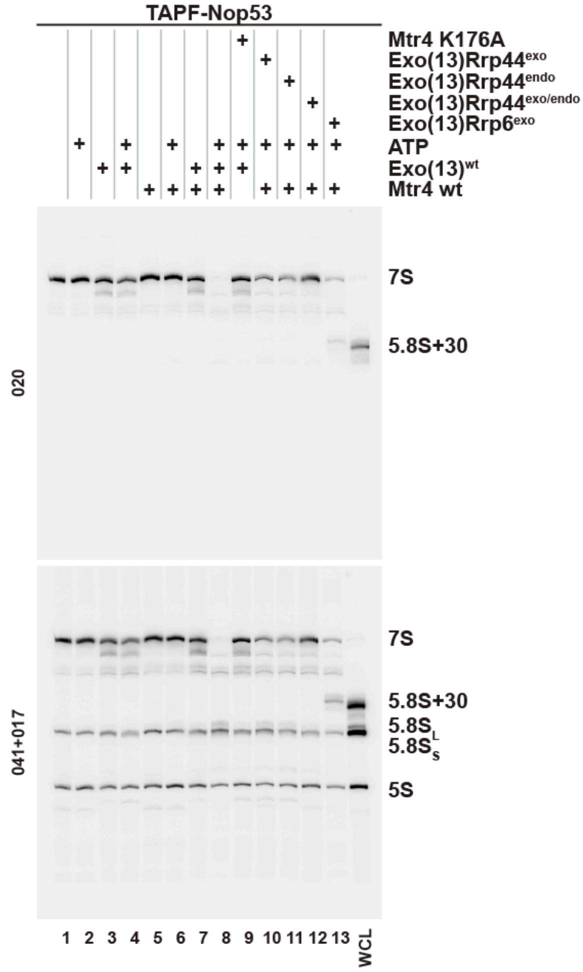

related to Fig. 2 a

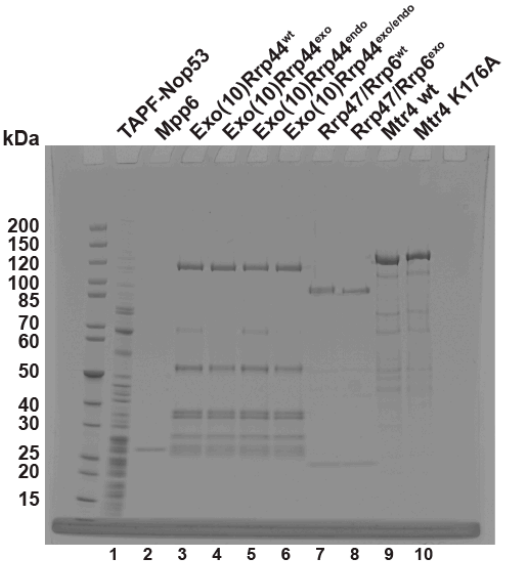

related to Fig. 3 a

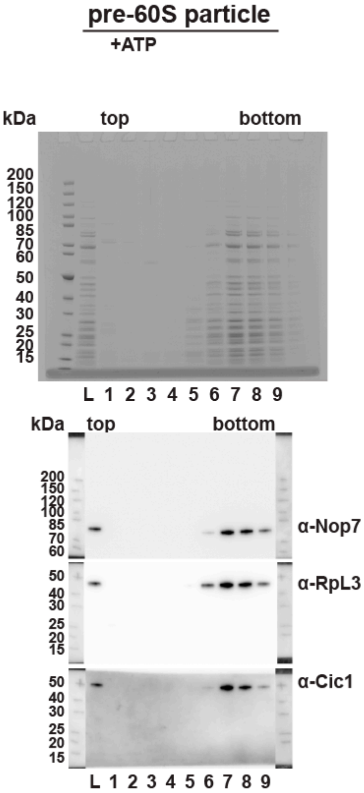

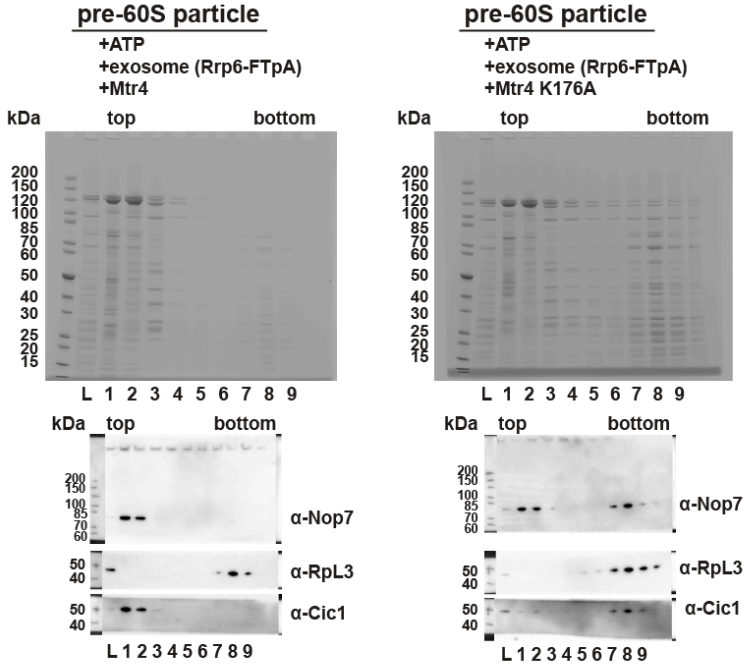

related to Fig. 5 b

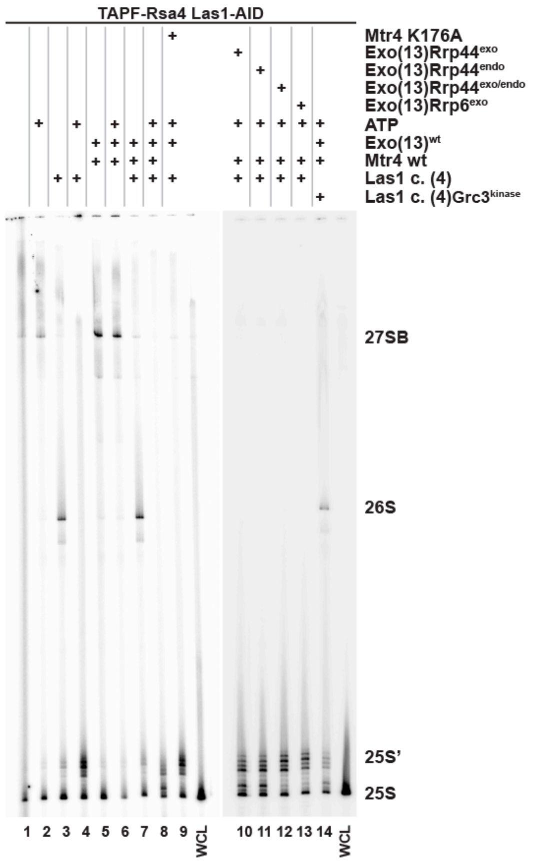

related to Fig. 5 a

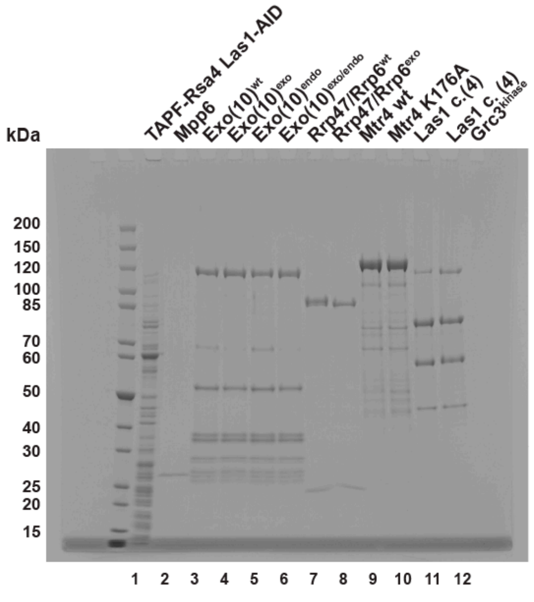

related to Fig. 5 c

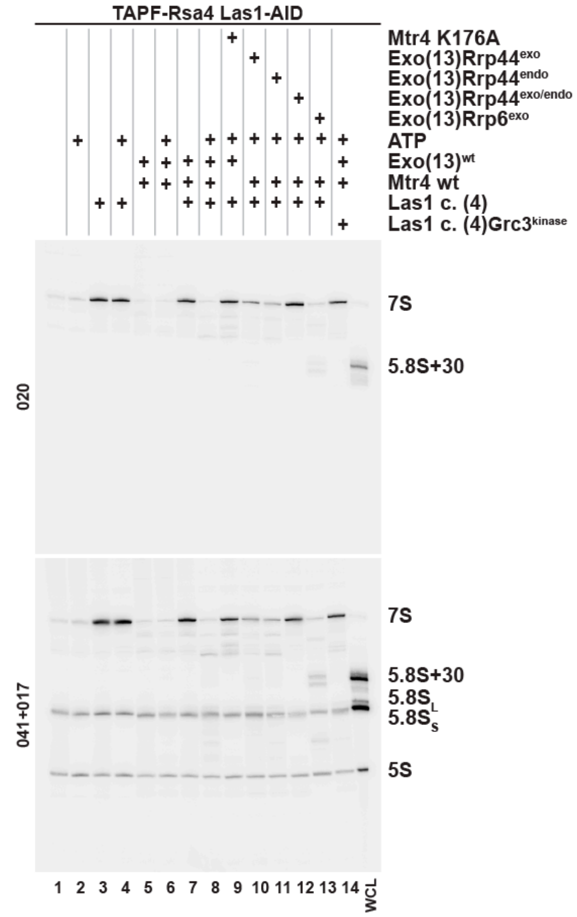

Supplement: Supplementary file 1 — Supplementary Information [file 41467_2017_1786_MOESM1_ESM.pdf]
